# A 12-Month Follow-Up of PROCARE+, a Transdiagnostic, Selective, Preventive Intervention for Adolescents At-Risk for Emotional Disorders

**DOI:** 10.1007/s10578-023-01638-2

**Published:** 2023-12-21

**Authors:** Manuel Vivas-Fernandez, Luis-Joaquin Garcia-Lopez, Jose A. Piqueras, Lourdes Espinosa-Fernandez, Jose-Antonio Muela-Martinez, David Jimenez-Vazquez, Maria del Mar Diaz-Castela, Jill Ehrenreich-May

**Affiliations:** 1 Department of Psychology, Division of Clinical Psychology, University of Jaen, Campus de Las Lagunillas S/N, C-5, Jaen, Spain; 2 Department of Health Psychology, Miguel Hernandez University, Elche, Spain; 3 Department of Psychology, University of Miami, Miami, USA

**Keywords:** Adolescence, Anxiety, Depression, Prevention, Transdiagnostic

## Abstract

Few studies have reported long-term follow-up data on selective preventive interventions for adolescents. No follow-up selective preventive transdiagnostic studies for adolescents at-risk for emotional disorders, such as anxiety and depression, have been reported. To fill this gap, this study aims to provide the first follow-up assessment of a randomized controlled trial (RCT) studying selective transdiagnostic prevention in at-risk adolescents. A 12-month follow-up assessment was conducted with subjects who originally received either PROCARE (Preventive transdiagnostic intervention for Adolescents at Risk for Emotional disorders), PROCARE+, which includes the PROCARE protocol along with personalized add-on modules or an active control condition (ACC) based on emotional psychoeducation, and their respective booster session for each experimental condition. 80 subjects (47.5% girls) aged between 12 and 18 years (M = 14.62; SD 1.43) who completed these treatment conditions were available for the 12-month follow-up. The results demonstrate the superior long-term efficacy of the PROCARE+ intervention in mitigating emotional symptoms and obsessive–compulsive symptomatology compared to the PROCARE and ACC conditions, with effect sizes notably exceeding those commonly observed in preventive programs. While the three treatments demonstrated beneficial impacts, the pronounced results associated with PROCARE+ at the 12-month follow-up emphasized the importance of personalized treatment modules and the sustained benefits of booster sessions in the realm of preventive psychological interventions. The findings also highlight the potential role of add-on modules in enhancing the effects of the PROCARE+ condition.

## Introduction

Approximately 50% of mental health problems have their onset before the age of 14, and 75% before the age of 18 [1, 2]. These alarming rates indicate with clarity that the timing of good mental health preventive interventions is as early as possible, but especially better before the age of 18 years [2–4].

More specifically, emotional disorders, which encompass a range of conditions primarily characterized by disturbances in mood, emotional regulation, and response to fear, are most prevalent among adolescents. Defined particularly by conditions like anxiety and depression, they often manifest as prolonged feelings of distress, sadness, or heightened anxiety. It is during adolescence that the initial symptoms or full-blown episodes of these disorders frequently emerge [2, 5, 6]. The challenges posed by the Covid-19 pandemic have further exacerbated this, leading to a notable rise in prevalence and an increased risk of adolescents suffering from these emotional disturbances [7–11].

The presence of clinical and subclinical disorders can potentially be a public health problem since they are negatively related to social and family malfunctioning, psychological distress, poor academic performance and increased suicidality [12–17]. The World Health Organization has stressed the need to prevent the consequences and the risk of chronicity of this problem, being necessary preventive interventions, prioritizing non-pharmacological approaches, with the aim of reinforcing the capacity of adolescents to regulate emotions, particularly among the most vulnerable [18, 19]. It is for this reason that selective prevention treatments are beneficial [20]. Selective prevention targets groups or individuals who exhibit specific risk factors, increasing their likelihood of developing symptoms compared to the broader population. This approach aims to address and mitigate these disorders before they escalate or become chronic. It is suggested that the positive effects of prevention programs diminish over time [21], so booster sessions are essential to maintain gains, as evidenced by studies that analyze their effect, making interventions that include them more effective and with larger effect sizes [22, 23]. Additionally, recent research supports the idea that group therapies often outperform individual sessions, allowing participants to gain insight from others’ experiences, promote mutual support, and foster a sense of community [24, 25]. The shift to online therapy formats has also shown promising results. Not only do they offer convenience and accessibility, but studies indicate that online interventions retain effectiveness, with some even suggesting that the digital format enhances patient engagement and reduces drop-out rates [26, 27].

PROCARE+ is a brief, personalized protocol for the selective prevention of emotional problems, adapted from the Unified Protocol for Transdiagnostic Treatment of Emotional Disorders in Adolescents (UP-A; [28] which includes additional modules tailored to the specific needs of each adolescent. This approach has been shown to be effective for the treatment of clinical disorders related to anxiety and depression in young population, as well as for universal and indicated prevention purposes [23, 28–35]. Vivas-Fernandez et al. [36], was the first approach in the transdiagnostic selective prevention of emotional disorders in the adolescent population, proving that PROCARE+ can reduce the risk of developing emotional disorders and anxious and depressive symptomatology, as well as it can increase resilience and emotion regulation after intervention and 6-month follow-up. In addition, recent systematic reviews concluded that there is a lack of selective prevention treatments focused on anxiety and depression in adolescents [37].

Bearing all of this in mind, this paper aims to examine the long-term effect of PROCARE+, PROCARE and an active control condition based on emotional psychoeducation (ACC), 12 months after the interventions. To further understand the long-term impact and sustainability of well-founded and evidence-based selective prevention programs with a transdiagnostic focus for adolescents at risk of emotional disorders, this 12-month follow-up study aims to: assess the sustained acceptability, fidelity, and adherence to the three interventions; measure the enduring effects of each of the three treatments in terms of emotional risk, defined as the likelihood of developing emotional disturbances due to stressors or adverse events, resilience, and quality of life related to physical, mental, and social health as primary outcomes; and track the continued development or maintenance of emotional regulation skills, cognitive flexibility, and anxiety and depression symptomatology as secondary outcomes over a 12-month period. More specifically, this follow-up evaluates the lasting efficacy of a CBT transdiagnostic selective prevention intervention, adapted from UP-A to 8 sessions and conducted in a telepsychology group format. This intervention included add-on modules to target adolescents’ specific needs and risk factors, termed PROCARE+, in comparison to the core intervention without add-on modules (PROCARE) and the aforementioned ACC. The booster session, administered 6 months post-intervention, is also examined for its potential to maintain benefits over the extended period.

## Material and Methods

### Participants

**Fig. 1:**
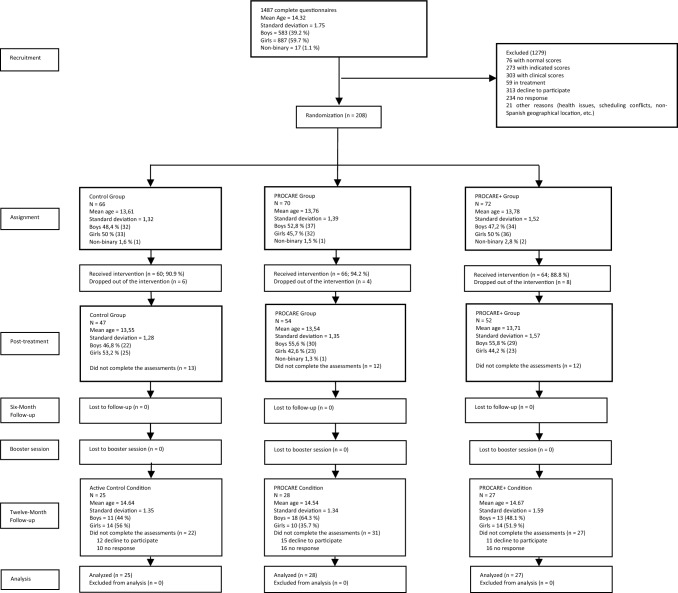
Consort flow diagram

Out of the initial sample (see Fig. 1), 80 adolescents (52.5% boys and 47.5% girls) aged between 12 and 18 years (M = 14.62; SD 1.43) were evaluated at follow-up 12 months after the initial intervention: ACC, n = 25 (53.19% of the sample retained); PROCARE, n = 28 (51.85% of the sample retained); and PROCARE+, n = 27 (51.92% of the sample retained). Each family, made up of the adolescent and their parents, was compensated with €50 for their active participation and time spent on the evaluations.

The inclusion criteria for the initial RCT were as follows: (1) having the informed consent of the adolescent and his or her guardian or legal custodian, (2) the technological means to attend the online sessions; (3) possible risk of emotional problems reported by the Spanish version of the emotional symptoms subscale of the Strengths and Difficulties Questionnaire (SDQ) in the Self-Reported or the Parent-Reported version [38, 39] (4) low or medium resilience reported by the *10-Item Connor-Davidson Resilience Scale* (CD-RISC-10) [40, 41], (5) low overall emotional symptomatology or scores below normative data for any of the subscales (depression, panic, social phobia, separation, generalized anxiety and obsessive compulsive disorder measured with the Revised Children’s Anxiety and Depression Scale (RCADS-30; [42, 43], (6) presence of at least one risk factor (social exclusion, stress-related situations, unhealthy lifestyle habits, parental-child interaction), (7) not receiving psychological or psychiatric treatment; (8) not presenting acute suicidality and (9) absence of neurodevelopmental disorders.

As can be seen in Table 1, age, gender and sociodemographic distribution was homogeneous among conditions and there were no statistical differences (*p* > 0.05).

**Table 1:** Socio-demographic variables

	ACC *M* (*SD*)	PROCARE *M* (*SD*)	PROCARE + *M* (*SD*)	
*N*	25	28	27	*ns*
Age	14.64 (1.35)	14.54 (1.34)	14.67 (1.59)	*ns*
Attendance (0–8)	7.64 (0.75)	7.79 (0.49)	7.59 (0.63)	*ns*
	*N*	*N*	*N*	
Gender				
Girls	14 (56%)	10 (35.7%)	14 (51.9%)	*ns*
Boys	11 (44%)	18 (64.3%)	13 (48.1%)	*ns*
Nationality				
Spanish	22 (88%)	22 (78.6%)	24 (88.9%)	*ns*
Non-Spanish	3 (12%)	6 (21.4%)	3 (11.1%)	*ns*

*M* (mean); *SD* (standard deviation); *ns* = non-significant *p* < .05

### Measures

In the assessment protocol, measures were categorized into primary and secondary outcomes, with every instrument being self-reported. Further details on these measures can be found in Vivas-Fernandez et al. [36].

Primary outcomes were assessed using the following measures: Emotional Risk subscale of *The Strengths and Difficulties Questionnaire* (SDQ; [44], with versions self-reported by both parents and adolescents, the *10-Item Connor-Davidson Resilience Scale* (CD-RISC-10; [40]. and the *KIDSCREEN-10 Index* [45], which measures quality of life.

Secondary measures included: *Difficulties in Emotion Regulation Scale* (DERS; [46], *Willingness & Action Measure for Children and Adolescents* (WAM-C/A; [47, 48], and *The Revised Child Anxiety and Depression Scale, 30-item version* (RCADS-30; [42]. Finally, to identify individual putative risk factors evidenced by adolescents, the following measures were taken: *Cyberbullying and bullying scale* [49], *Fear of COVID-19 Scale* (FCV-19S; [50], *Structured Interview for the Assessment of Expressed Emotion: Child version* (E5cv; [51] and an ad-hoc 9-questions questionnaire designed to detect unhealthy lifestyle habits.

### Procedure

The present study is a 12-month follow-up of the research study of Vivas-Fernandez et al. [36] that follows a 3-arm randomized controlled trial (Arm 1 = ACC, Arm 2 = PROCARE; Arm 3 = PROCARE+) in the Spanish adolescent population. For a detailed understanding of the procedure, please refer to the article. The initial sample was recruited through secondary education centers, social media, mass press, as well as local, regional and national administrations of the Spanish state organization related to education and youth.

Informed consent was obtained from the legal guardians and the adolescents themselves, and all assessments were performed in an online format through a secure platform. The initial sample of adolescents was randomly allocated to the ACC, PROCARE and PROCARE+ conditions without prior knowledge of the specific telehealth-based treatment they would receive. An intention-to-treat analysis revealed no significant differences (*p* > 0.05) between the initial sample and the 12-month retained sample. 153 adolescents (ACC, n = 47; PROCARE, n = 54; and PROCARE+, n = 52) received the primary intervention and were assessed at post-treatment and at 6-month follow-up. They subsequently received an online booster session and were evaluated 1 month after that session (7 months after the main intervention). The youth booster session consisted of a 90-min session aimed at reviewing and refreshing participants’ acquired skills during the course.

During the 12-month follow-up assessment periods, all parents and adolescents were reassessed with the primary and secondary measures. Following the recommendations of EU Clinical Trial Directive (2001/20/EC) and Regulation (536/2014), only adolescents and their parents who took part in the follow-up evaluations were eligible to receive a €50 compensation for their time.

### Interventions

The three experimental conditions (PROCARE, PROCARE+ and ACC) included a 15-min initial informational and individual session with the adolescent and their family (session 0). The following sessions were online group-based (6–8 adolescents) sessions run by a therapist and a co-therapist certified by the University of Miami. Fidelity sheets were filled in by therapists after each session and were supervised to maintain maximum adherence to the treatment content and manual instructions. The three conditions included a booster session to maintain the effects of the interventions over time. Each youth and parent booster session consisted of a 90-min session delivered 6 months after intervention, after the 6-month follow-up. Specifically, these sessions were designed to provide a comprehensive recap of the content covered in each workshop session, aimed at consolidating the skills acquired by the participants during the program. Parents’ roles were primarily informational: they received updates after each session detailing the content and activities. However, parents who were specifically delegated to the additional parents’ module in the PROCARE+ condition played a more proactive role in the intervention process. Parents who were specifically delegated to the additional parents’ module in the PROCARE+ condition played a more proactive role in the intervention process.

The PROCARE condition was an 8-session adaptation of the Unified Protocol for Transdiagnostic Treatment of Emotional Disorders in Adolescents (UP-A; [28]. It uses evidence-based CBT strategies targeting emotional disorders. Focuses on improving emotional reactivity, regulation, and distress tolerance in adolescents. Modules include emotion education, emotion-focused experiments, awareness of sensations, flexible thinking, emotional awareness, and situation-based emotion exposures.

The PROCARE+ condition included PROCARE as well as additional modules for adolescents and parents, tailored according to the risk factor evidenced by the adolescents. The add-on youth module sessions were conducted in smaller groups of 5, 6 participants and included three modules for adolescents (social exclusion, Covid-19 stress-related and healthy habits through 1-h length therapeutic sessions). Furthermore, there was a module for parents (to improve parent–child communication skills with a particular emphasis on reducing levels of parental expressed emotion). Adolescents benefitted from one or more add-on modules, depending on their risk factors. Parents with a high level of expressed emotion were eligible for the parental add-on module.

The ACC was an abbreviated 8-week adaptation of the 12-week Utalk intervention, delivered in group format [52]. UTalk provides psychoeducation on how emotions work and includes discussion groups where adolescents talk about how they experience their emotions. No explicit coping strategies to deal with strong emotions or CBT strategies beyond psychoeducation were provided as part of this program.

For a detailed overview of the interventions, please refer to Vivas et al. [36].

### Data Analysis

Data were coded and analyzed with the statistical package IBM SPSS Statistics 28.0 [53]. First, the retained sample at the 12-month follow-up was analyzed to ensure that primary and secondary outcomes in the pretest of the three conditions were similar to those of the initial sample. Analyses were carried out to ensure the equivalence of the experimental conditions. Comparisons between sociodemographic variables between ACC, PROCARE and PROCARE+ were calculated by means of *Z* (Mann–Whitney *U* test) for nonparametric comparisons with a quantitative variable; *χ*^2^ (Chi-square) for comparisons with a nominal variable and *LR* (Likelihood Ratio) for comparison with a nominal variable when at least 80% of the expected frequencies were < 5). As no differences were found according to any of the sociodemographic variables (*p* > 0.05), subsequent analyses were performed without controlling for gender, age or any other variables. Between-group analysis were examined using Kruskal Wallis test at the 12-month follow-up, due to non-compliance of normality assumptions [54]. Subsequently, for the comparisons that were significant, a post hoc analysis was performed using the Mann–Whitney *U* test. Finally, within-group comparisons were calculated for each condition. Comparisons between pre-test, post-test, 6-months follow-up and 12-months follow-up were computed, using the Student’s *t*-test paired-samples or the Wilcoxon signed-rank test (for nonparametric comparisons). Effect sizes were analyzed using the Cohen’s *d* and Pearson’s *r* [55]. For parametric comparisons, Cohen’s *d*: small (a1) = 0.2, medium (a2) = 0.5, large (a3) = 0.8 was used; for nonparametric comparisons Pearson’s *r*: small (b1) = 0.1, medium (b2) = 0.3, large (b3) = 0.5.

## Results

### Between-Group Analyses

No statistically significant differences were found in the sociodemographic variables between the conditions regarding age, (*Z* = 0.19, *p* = 0.90), gender (*χ*^2^ (2) = 1.54, *p* = 0.46), nationality (*LR* (2) = 1.06, *p* = 0.58) or attendance to sessions (*Z* = 2.06, *p* = 0.35).

#### Primary Outcomes

As can be seen in Table 2, global between-group analyses revealed significant differences in the level of emotional risk, with small effect size, as measured by the self-reported (*H* (2) = 0.09, *p* = 0.03) and parent-reported (*H* (2) = 0.09, *p* = 0.02) emotional problems subscale of the SDQ. Post-hoc comparisons between ACC and PROCARE conditions (see Table 3) did not show significant differences in the primary outcomes. Post-hoc comparisons between ACC and PROCARE+ found that the latter evidenced significant improvements compared to the former in the reduction of level of emotional risk reported by parents (*Z* = − 2.19, *p* = 0.01) and adolescents (*Z* = − 2.55, *p* = 0.02) with medium effect sizes. Finally, comparison between PROCARE and PROCARE+ revealed that PROCARE+ was significantly superior in the reduction of level of emotional risk reported by parents (*Z* = − 2.34, *p* = 0.05) and adolescents (*Z* = − 1.89, *p* = 0.01) with medium to large effect sizes.

**Table 2:** Global between-group comparisons (ACC, PROCARE, PROCARE+)

Measures	12-month follow-up mean (SD)						Effect size (Pearson’s r)
	ACC		PROCARE		PROCARE+		
Primary outcome measures	Median	Range	Median	Range	Median	Range	
Self-report SDQ	2	10	2	6	1	5	0.09_b1_*
Parent SDQ	2	7	2	6	1	4	0.09_b1_*
CD-RISC	29	33	30	23	31	20	
KIDSCREEN	35	23	37,5	25	39	18	
Secondary outcome measures							
DERS	67	93	72,5	68	61	62	
WAM	41	48	45	46	45	41	
RCADS (Total)	16	54	14,5	36	14	43	
RCADS (GAD)	4	14	5	14	4	11	
RCADS (SoP)	4	15	4,5	11	3	8	
RCADS (PD)	1	12	0	5	0	8	
RCADS (MDD)	3	10	3	6	2	8	
RCADS (SAD)	0	8	0	4	0	7	
RCADS (OCD)	3	8	1,5	9	1	8	0.08_b1_*

*Self-Report SDQ* The Strengths and Difficulties Questionnaire (Adolescents. Emotional Problems Subscale); *Parent SDQ* The Strengths and Difficulties Questionnaire (Parents) Emotional Problems Subscale, *CD-RISC* 10-Item Connor-Davidson Resilience, *KIDSCREEN* KIDSCREEN-10 Index, *DERS* Difficulties in Emotion Regulation Scale, *WAM* willingness & action measure for children and adolescents, *RCADS (Total)* Revised Children’s Anxiety and Depression Scale. Total score; *RCADS (GAD)* RCADS Generalized Anxiety Disorder Subscale, *RCADS (SoP)* RCADS Social Phobia Subscale, *RCADS (PD)* RCADS Panic Disorder Subscale, *RCADS (MDD)* RCADS Major Depressive Disorder Subscale, *RCADS (SAD)* RCADS Separation Anxiety Disorder Subscale, *RCADS (OCD)* RCADS Obsessive Compulsive Disorder Subscale

Effect size: Pearson’*r* (non-parametric): small (b1) = 0.1, medium (b2) = 0.3, large (b3) = 0.5

*p ≤ 0.05

**p ≤ 0.01

***p ≤ 0.001

**Table 3:** Post-hoc comparisons

Measures	ACC vs PROCARE (PR) Effect size (Pearson’s *r*)	ACC vs PROCARE + (PR+) Effect size (Pearson’s *r*)	PROCARE (PR) vs PROCARE + (PR+) Effect size (Pearson’s *r*)
	12-month follow-up	12-month follow-up	12-month follow-up
Primary outcome measures			
Self-Report SDQ		ACC < PR + 0.30_b2_*	PR < PR + 0.32_b2_*
Parent SDQ		ACC < PR + 0.35_b2_*	PR < PR + 0.27_b1_*
CD-RISC			
KIDSCREEN			
Secondary outcome measures			
DERS			
WAM			
RCADS (Total)			
RCADS (GAD)			
RCADS (SoP)			
RCADS (PD)			
RCADS (MDD)			
RCADS (SAD)			
RCADS (OCD)	ACC < PR 0.32_b2_*	ACC < PR + 0.30_b2_*	

*Self-Report SDQ* The Strengths and Difficulties Questionnaire (Adolescents. Emotional Problems Subscale), *Parent SDQ* The Strengths and Difficulties Questionnaire (Parents) Emotional Problems Subscale, *CD-RISC* 10-Item Connor-Davidson Resilience, *KIDSCREEN* KIDSCREEN-10 Index, *DERS* Difficulties in Emotion Regulation Scale, *WAM* willingness & action measure for children and adolescents, *RCADS (Total)* Revised Children’s Anxiety and Depression Scale. Total score; *RCADS (GAD)* RCADS Generalized Anxiety Disorder Subscale, *RCADS (SoP)* RCADS Social Phobia Subscale, *RCADS (PD)* RCADS Panic Disorder Subscale, *RCADS (MDD)* RCADS Major Depressive Disorder Subscale, *RCADS (SAD)* RCADS Separation Anxiety Disorder Subscale, *RCADS (OCD)* RCADS Obsessive Compulsive Disorder Subscale

Effect size: Pearson’*r* (non-parametric): small (b1) = 0.1, medium (b2) = 0.3, large (b3) = 0.5

*p ≤ 0.05

**p ≤ 0.01

***p ≤ 0.001

#### Secondary Outcomes

Regarding global between-group comparisons for secondary outcomes, significant differences were limited to the obsessive–compulsive RCADS subscale, with small effect sizes (*H* (2) = 0.87, *p* = 0.03). Post-hoc comparisons between ACC and PROCARE conditions indicated PROCARE was significantly superior in the reduction of obsessive–compulsive symptoms with medium effect sizes (*Z* = − 2.20, *p* = 0.02). The comparison between ACC and PROCARE+ revealed that the latter was significantly superior in the reduction of level of symptomatology for obsessive–compulsive disorder (*Z* = − 2.20 *p* = 0.02), with medium effect size. Post- hoc comparisons between PROCARE and PROCARE+ conditions did not show significant improvements for any secondary outcomes.

### Within-Group Analyses

#### Primary Outcomes

Significant differences for ACC were limited to the level of emotional risk reported by parents, as measured by the SDQ, between the pretreatment and the 12-month follow-up (see Table 4), with large effect sizes (*t* = 3.12, *p* = 0.005) and to the quality of life, as measured by the KIDSCREEN-10, between the post-treatment and the 12-month follow-up, with medium effect sizes (*t* = 2.37, *p* = 0.02). Significant differences for PROCARE were limited to the reduction of level of emotional risk as reported by parents, when 12-month follow-up data were compared to pre-treatment (*Z* = − 3.69, *p* = 0.00), post-treatment (*Z* = − 2.62, *p* = 0.00) and 6-months follow-up (*Z* = − 2.24, *p* = 0.02), with effect sizes ranging from small to large. Moreover, resilience improved between the pretest and the 12-month follow-up with large effect sizes (*t* = − 3.16, *p* = 0.00). Within-group analysis for the PROCARE+ condition revealed improvements between the pre-treatment and the 12-month follow-ups in all primary outcome measures. Effect sizes ranged from small to large.

**Table 4:** Within-group comparisons

Measures		Baseline/pre-treatment		Post-treatment		6-month F-U		Post-booster		12-month F-U		Effect size (Cohen’s *d*/Pearson’s *r*)			
Primary outcome measures		Median	Range	Median	Range	Median	Range	Median	Range	Median	Range	Pre-treatment-12-months follow-up	Post-treatment-12-months follow-up	6-months Follow-Up-12-months follow-up	Post-booster-12-months follow-up
Self-Report SDQ	ACC	3	6	3	6	2	7	2	8	2	10				
	PROCARE	3	6	2	9	2	6	1.5	6	2	6				
	PROCARE+	2	5	1	5	2	5	1	4	1	5	0.62_b3_***			
Parent SDQ	ACC	3	9	1	7	2	6	2	7	2	7	0.62a2**			
	PROCARE	3	9	3	5	2	6	2	5	2	6	0.69_b3_***	0.49_b2_**	0.42_b2_*	
	PROCARE+	2	8	1	5	1	6	1	4	1	4	0.63_b3_***			
CD-RISC	ACC	27	24	29	24	28	21	28	22	29	33				
	PROCARE	24.5	16	27	40	27	40	27	23	28	23	0.59_b3_**			
	PROCARE+	26	21	29	37	30	20	32	16	31	20	0.92_b3_***			
KIDSCREEN	ACC	37	19	38	20	37	26	36	23	35	23		0.47_a1_*		
	PROCARE	36	16	38	30	39	23	38.5	27	37.5	25				
	PROCARE+	37	16	38	24	38	18	40	18	39	18	0.51_a2_**			
Secondary outcome measures															
DERS	ACC	89	81	68	90	75	71	74	81	67	93				
	PROCARE	79	88	71	88	70	82	70	88	72.5	68	0.50_b3_*			
	PROCARE+	75	90	73	56	67	67	68	66	61	62	0.55_a1_**			
WAM	ACC	41	34	45	43	47	45	44	43	41	48				
	PROCARE	39.5	28	41.5	41	45	41	44	50	45	46	0.45_a1_*			
	PROCARE+	39	43	42	45	40	49	44	34	45	41	0.53_a2_**		0.48_a1_*	
RCADS (Total)	ACC	22	30	21	40	24	46	23	46	16	54				
	PROCARE	24	32	18	51	19	49	16	43	14.5	36	0.68_a2_***		0.62_a2_**	
	PROCARE+	19	35	15	37	17	41	15	49	14	43	0.51_a2_**		0.69_b3_***	
RCADS (GAD)	ACC	6	11	5	13	6	15	4	15	4	14				
	PROCARE	6.5	11	5	11	7	14	5	9	5	14	0.43_a1_*		0.39_a1_*	
	PROCARE+	6	8	5	10	5	12	5	9	4	11	0.51_a2_**		0.39_a1_*	
RCADS (SoP)	ACC	5	11	5	14	5	13	5	15	4	15				
	PROCARE	5.5	15	4	15	5	15	4.5	15	4.5	11	0.47_a1_*		0.41_b2_*	
	PROCARE+	5	15	4	12	5	9	4	10	3	8	0.60_a2_**		0.67_a2_**	0.40_a1_*
RCADS (PD)	ACC	2	6	1	7	1	8	1	11	1	12				
	PROCARE	1	4	1	6	1	6	0	9	0	5		0.38_b2_*	0.47_b2_*	
	PROCARE+	1	5	0	5	1	4	0	4	0	8	0.37_b3_*			
RCADS (MDD)	ACC	3	8	3	9	4	12	4	8	3	10				
	PROCARE	4	8	3	11	3	9	3	9	3	6				
	PROCARE+	3	8	2	8	2	6	2	7	2	8				
RCADS (SAD)	ACC	1	6	0	6	0	9	0	8	0	8				0.54_b3_**
	PROCARE	1	7	1.5	4	0	8	0	6	0	4	0.46_b3_*	0.50_b3_**		
	PROCARE+	0	6	0	5	0	8	0	8	0	7				
RCADS (OCD)	ACC	3	8	3	6	3	11	3	8	3	8				
	PROCARE	3	9	2	11	2.5	8	2	9	1.5	9	0.58_b3_**		0.44_b2_*	
	PROCARE+	2	8	3	7	2	8	2	9	1	8	0.36_b3_*			

*Self-Report SDQ* The Strengths and Difficulties Questionnaire (Adolescents. Emotional Problems Subscale), *Parent SDQ* The Strengths and Difficulties Questionnaire (Parents) Emotional Problems Subscale, *CD-RISC* 10-Item Connor-Davidson Resilience, *KIDSCREEN* KIDSCREEN-10 Index, *DERS* Difficulties in Emotion Regulation Scale, *WAM* Willingness & Action Measure for Children and Adolescents, *RCADS (Total)* Revised Children’s Anxiety and Depression Scale. Total score; *RCADS (GAD)* RCADS Generalized Anxiety Disorder Subscale, *RCADS (SoP)* RCADS Social Phobia Subscale, *RCADS (PD)* RCADS Panic Disorder Subscale, *RCADS (MDD)* RCADS Major Depressive Disorder Subscale, *RCADS (SAD)* RCADS Separation Anxiety Disorder Subscale, *RCADS (OCD)* RCADS Obsessive Compulsive Disorder Subscale

Effect size: Cohen’s *d* (parametric): small (a1) = 0.2, medium (a2) = 0.5, large (a3) = 0.8

Effect size: Pearson’s *r* (non-parametric): small (b1) = 0.1, medium (b2) = 0.3, large (b3) = 0.5

*p ≤ 0.05.; **p ≤ 0.01; ***p ≤ 0.001

#### Secondary Outcomes

Within-group analysis for ACC revealed significant differences between the post-booster and the 12-month follow-up in separation anxiety symptomatology (*Z* = − 2.70, *p* = 0.00), with large effect size.

Within-group analysis for PROCARE showed significant differences between the pretreatment and the 12-month follow-up in most secondary measures with effect sizes ranging from small to large. Statistical differences between the 6 and 12-month follow-ups were found in all subscales measured by the RCADS, with the exception of depressive and separation anxiety symptomatology, with small to medium effect sizes.

Within-group analysis for the PROCARE+ condition revealed differences between the pretreatment and the 12-month follow-up in all secondary measures (see Table 4). Effect sizes ranged from small to large. Statistical differences between the 6-month follow-up and the 12-month follow-up in all secondary measures with the exception of depressive and separation anxiety symptomatology. Effect sizes ranged from small to large. Statistical differences between the 6-month follow-up and the 12-month follow-up included most of the secondary measures except for emotion regulation, panic, depressive, separation anxiety and obsessive–compulsive symptomatology. Effect sizes ranged from small to large. Additionally, significant improvements in social anxiety symptomatology were found between post-booster and 12-month follow-up, with small effect size (*t* = 3.48, *p* = 0.04).

## Discussion

This study was aimed at examining the long-term efficacy of three interventions for adolescents at-risk for emotional problems. Between-group comparisons at 12-month follow-up showed significant differences across conditions both in the risk of emotional symptoms and obsessive–compulsive symptomatology, with small effect sizes. Meta-analytic reviews suggest that prevention programs for depression and anxiety have small to medium effect sizes [56, 57]. The effect sizes found in this study are in line with what is expected for this population.

Between-group post-hoc analyses at 12-month follow-up revealed that PROCARE was significantly superior to the ACC condition but limited to obsessive–compulsive symptomatology, with medium effect sizes. This special effect of transdiagnostic interventions in decreasing obsessive–compulsive symptomatology has been evidenced in other studies [58, 59]. Furthermore, PROCARE+ was superior to ACC in the emotional risk identified by both adolescents and parents and, as secondary outcome, on obsessive–compulsive symptomatology, with medium effect sizes. These findings are in line with other studies which have analyzed the long-term effect of transdiagnostic treatments focused on clinical disorders [28, 56, 57], including indicated transdiagnostic prevention treatments [23, 33, 35]. Finally, PROCARE+ was superior to PROCARE, with significant differences in the risk of emotional problems identified by both the adolescents and parents, ranging from small to medium effect sizes. The effect of PROCARE+ over PROCARE, limited to the inclusion of personalized add-on modules, suggests the importance of modules tailored to the adolescents’ needs in order to further reduce the level of emotional risk. These results are consistent with other studies pointing out the importance of personalized medicine in psychological interventions [9, 10, 60] and aligned with promising research on personalized medicine in which modest effect sizes have been found in cognitive-behavioral treatments controlling the effect of treatment moderators [61].

In particular, within-group comparisons between the pre-treatment and the 12-month follow-up for ACC revealed no significant changes in most of the primary or secondary variables. These findings are in the same line as studies reporting that short-term gains from preventive interventions for anxiety and/or depression tend to decrease over time [21]. Significant differences in primary outcomes for ACC were limited to the reduction in parent-reported emotional risk, as measured by the SDQ, and the improvement of quality of life as measured by the KIDSCREEN-10. As for secondary measures, differences were only found in global anxiety-depressive symptoms and obsessive–compulsive symptomatology measured by RCADS. Significant differences between the post-booster and the 12-month follow-up were limited to separation anxiety. There were no differences between the 6- and 12-month follow-up. However, significant differences described above suggest a small treatment benefit effect, consistent with the previous open trial study conducted by LaGreca et al. [47].

As far as within-group comparisons for the PROCARE condition are concerned, significant differences were found in most variables between the pre-treatment and the 12-month follow-up, with similar results to those found after the completion of the main treatment [36] and providing evidence in line with promising previous studies of the transdiagnostic approach for young people with clinical, indicated and universal samples [23, 29–32, 34, 35, 62]. The sustained outcomes seen in PROCARE differ from research that shows a diminishing effect of preventive interventions as time goes on [21]. Within-group comparisons between the 6- and the 12-month follow-up showed PROCARE exhibited significant differences in the reduction of emotional symptomatology reported by parents, with medium effect size. The booster sessions held after 6 months of treatment seem to have a role in supporting emotional gains. Follow-up findings suggest a possible link between these sessions and sustained intervention effects, as well as a reduced likelihood of symptom relapse [57, 63]. Furthermore, in line with the recommendations by Gearing et al. [22], selective intervention programs for at-risk adolescents can produce small to medium beneficial effects when follow-up assessments and booster sessions are included, as observed in the PROCARE+ condition.

Finally, the PROCARE+ condition evidenced significant improvements in every primary and secondary outcome in comparisons between the pre-treatment and the 12-month follow-up. This means that there has been a decrease in the risk of developing an emotional problem, an improvement in resilience, quality of life, emotional regulation, cognitive flexibility and anxiety and depressive symptoms, maintaining the same gains found in previous phases of the study [36] and in contrast with research which concluded that the effect of preventive interventions tend to decrease over time [64, 65]. Furthermore, comparisons between the 6- and 12-month follow-up showed that PROCARE+ exhibited significant differences in the reduction of emotional symptomatology, as measured by the RCADS and its subscales, with medium to large effect sizes. Differences were also observed between the post-booster and the 12-month follow-up assessments but limited to social phobia symptomatology. The booster session conducted 6 months after the completion of the intervention may have a role in the maintenance of effects or reduction of the likelihood of symptoms relapse over time, just as was observed in the PROCARE condition [21, 66].

Overall, the three conditions evidenced positive impact on adolescents’ wellbeing. However, it must be noted that PROCARE+ showed a larger number of improvements in treatment outcome measures, with higher effect sizes at the 12-month follow-up, in line with the positive findings found by Vivas-Fernandez et al. [36]. These results highlight the importance of the personalized medicine which characterizes PROCARE+, where additional modules are added to address the specific risk factors for each adolescent, as recommended in previous studies [9, 10, 60, 67, 68]. The booster session’s effect is more pronounced in the PROCARE+ condition than in PROCARE, potentially because it includes extended material from additional modules unique to this intervention. This might explain the more substantial gains observed post-treatment in PROCARE+. Consequently, while improvements are seen in both groups after the booster session conducted 6 months later, they are especially marked in the PROCARE+ condition due to its personalized pretreatment having a heightened preventive effect [36]. Similar to Vivas-Fernandez et al., [36] study, it appears that the SDQ emotional subscale (self-reported and/or parent-reported measure) is an instrument particularly sensitive for selective prevention purposes. Studies have similarly highlighted the effectiveness of the SDQ both in clinical settings and as a tool to identify symptomatology geared towards prevention [69–71].

Some limitations should be noted. First, while an intention-to-treat analysis was conducted to ensure data validity, the study would have benefited from a larger sample. This was mainly due to a reduction in participants for the 12-month follow-up, as some opted not to continue with the assessment process. Second, as the trial was conducted during the Covid-19 pandemic, and it is possible that improvements observed may have been influenced by the passage of time or by the normalization of the social situation in relation to Covid-19. To provide a clearer understanding, future studies might consider using a wait-list control group to discern these effects more distinctly. Notably, research indicates that adolescents remain among the most emotionally affected groups post-pandemic. Third, the booster sessions, while potentially pivotal, were not randomized, and hence, no direct causality can be established. It remains uncertain whether differences in outcomes for PROCARE+ participants were mainly due to the number or specificity of additional modules they received. This underscores the question of whether the success of PROCARE+ can be attributed mainly to an increase in treatment dosage or is more closely tied to the specificity of the modules based on the personal risk profile. Exploring this aspect will be central in future research. Lastly, forthcoming studies should also delve into the cost-effectiveness of prevention programs, as well as potential mediators and moderators of treatment.

## Conclusions

This is the first time that a selective preventive transdiagnostic intervention was tested in a 12-month follow-up. The research emphasizes the long-term efficacy of targeted interventions for adolescents at risk for emotional disturbances, with a special focus on the success of the PROCARE+ approach. Benefits observed at the 12-month follow-up indicate that personalized, module-based treatments potentially outpace standard prevention programs in producing more significant and enduring improvements.

Notably, the PROCARE and PROCARE+ conditions exhibited significant differences 12 months post-treatment, pointing to a positive long-term impact on emotional health. The findings particularly spotlight the superiority of PROCARE+ over both PROCARE and ACC, underscoring its effectiveness in reducing the risk of emotional symptoms in at-risk adolescents over extended periods.

The booster sessions, introduced 6 months after the primary treatment, appear to enhance the interventions’ positive impact, emphasizing the importance of consistent, tailored support in consolidating treatment gains. Furthermore, add-on modules have emerged as particularly influential in the PROCARE+ condition, suggesting they play a pivotal role in diminishing emotional risk.

Additionally, the study reaffirms the SDQ emotional subscale’s consistent performance, underscoring its utility as a trusted instrument for evaluating emotional risks and the outcomes of preventive interventions.

## Summary

This study aimed to explore the long-term effects of PROCARE+, an intervention for adolescents at risk of developing emotional problems, by comparing it with two interventions: PROCARE and an active control condition (ACC) based on emotional psychoeducation. Focusing on a 12-month follow-up period, the study assessed the sustainability of these interventions in reducing emotional risk, increasing resilience and improving quality of life and emotional regulation. The PROCARE+ and PROCARE interventions were adapted from the Unified Protocol for the Transdiagnostic Treatment of Emotional Disorders in Adolescents (UP-A), with PROCARE+ providing additional modules tailored to individual needs.

The prevalence of emotional disorders in adolescents, particularly anxiety and depression, has been exacerbated in the context after the Covid-19 pandemic. These disorders are associated with a range of negative consequences, including social and family dysfunction, psychological distress, poor academic performance and increased suicidality. The importance of investing in non-pharmacological preventive interventions in this context, focusing on emotional regulation and resilience promotion, especially among vulnerable groups, has been highlighted.

The study findings indicated that while all three conditions resulted in positive outcomes in terms of adolescent well-being, PROCARE+ was the most effective. This intervention produced significant improvements in emotional risk, resilience, quality of life, emotional regulation, cognitive flexibility, and symptoms of anxiety and depression. The addition of personalised modules in PROCARE+ was particularly effective, underscoring the importance of personalised approaches in psychological interventions.

In comparison, PROCARE showed significant benefits, but these were more limited than PROCARE+. ACC showed the least improvement, indicating the value of designing more structured and comprehensive interventions such as PROCARE and PROCARE+. Booster sessions, conducted 6 months after the intervention, could play an important role in maintaining the benefits of the interventions over time.

In conclusion, this study supports the efficacy of CBT-based preventive interventions from the transdiagnostic approach for adolescents at risk of emotional disorders, tailored to their specific needs.

## Data Availability

Data sets generated and/or analyzed during this study are not publicly available due to organizational limitations, but are available from the corresponding author upon reasonable request.
